# Racemic (*RS*_C_,*SR*_S_)-(2-{[1-allyl­oxy­carbonyl-3-(methyl­sulfanyl)prop­yl]iminometh­yl}phenyl-κ^3^ *S*,*N*,*C* ^1^)chlorido­platinum(II)

**DOI:** 10.1107/S160053680904238X

**Published:** 2009-10-23

**Authors:** Katsuhiro Isozaki, Akira Sato, Kazushi Miki

**Affiliations:** a Organic Nanomaterials Center, National Institute for Materials Science, Tsukuba, Ibaraki 305-0044, Japan; b Materials Analysis Center, National Institute for Materials Science, Tsukuba, Ibaraki 305-0044, Japan; c Applied Scoences, University of Tsukuba, Tsukuba, Ibaraki 305-8571, Japan

## Abstract

The title compound, [Pt(C_15_H_18_NO_2_S)Cl], was obtained by the cyclo­metallation reaction of *cis*-bis­(benzonitrile)dichlorido­platinum(II) with *N*-benzyl­idene-l-methio­nine allyl ester in refluxing toluene. The Pt^II^ atom has a square-planar geometry and is tetra-coordinated by the Cl atom and the C, N and S atoms from the benzyl­idene methio­nine ester ligand. In the crystal structure, the S atoms show opposite chiral configurations with respect to the α-carbon of the methio­nine, reducing steric repulsion between the methyl and allyl ester groups.

## Related literature

For cyclometallated Pt^II^ complexes having terdentate benzylidenamine ligands cyclo­metallated benzyl­ideneamine, see: Capapé *et al.* (2005 ▶); Caubet *et al.* (2003 ▶); Riera *et al.* (2000 ▶). For organometallic amino acid complexes, see: Severin *et al.* (1998 ▶).
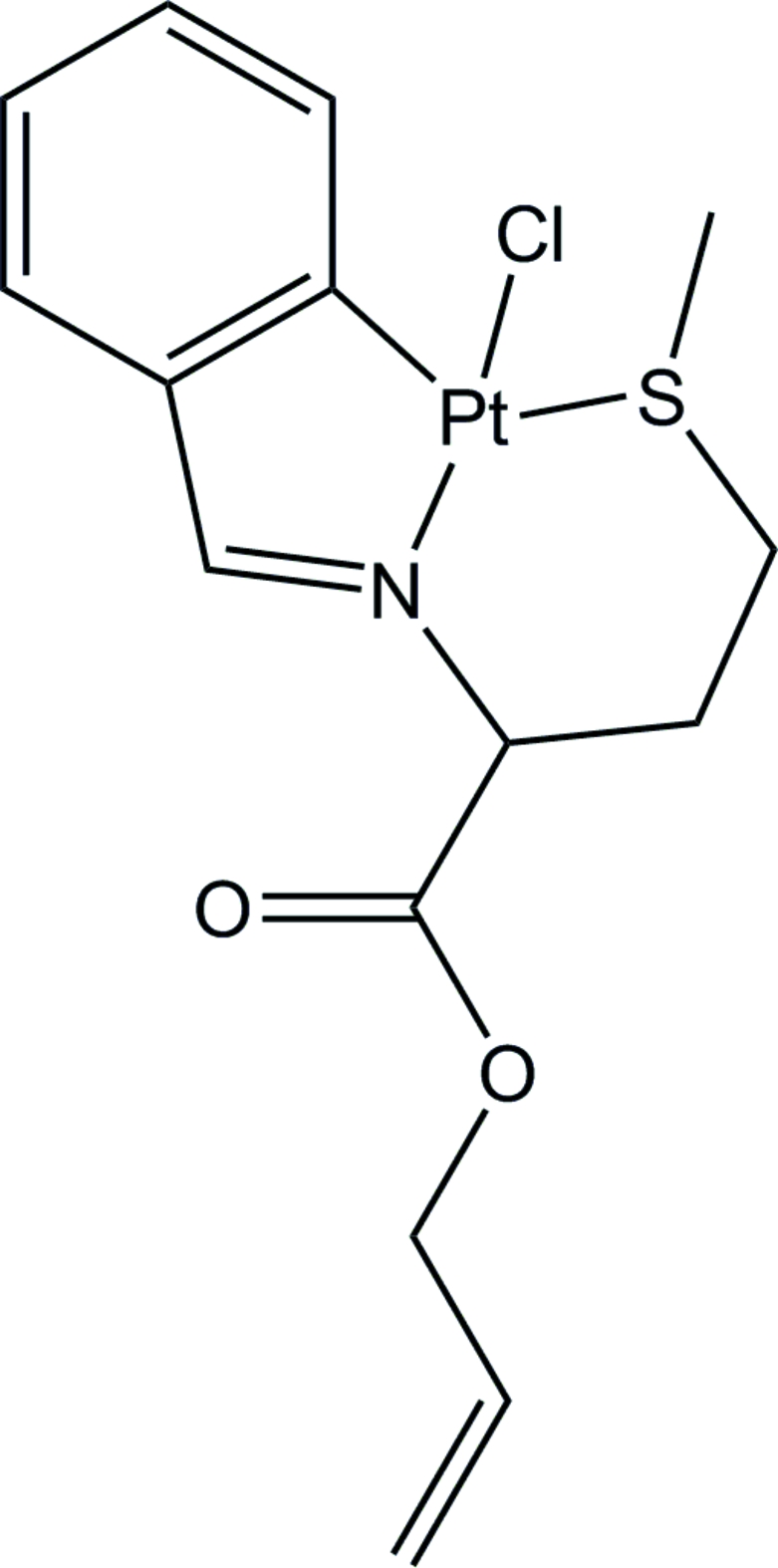


## Experimental

### 

#### Crystal data

[Pt(C_15_H_18_NO_2_S)Cl]
*M* *_r_* = 506.90
Monoclinic, 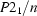

*a* = 8.6000 (13) Å
*b* = 9.5093 (14) Å
*c* = 19.679 (3) Å
β = 94.398 (2)°
*V* = 1604.6 (4) Å^3^
*Z* = 4
Mo *K*α radiation
μ = 9.04 mm^−1^
*T* = 180 K
0.50 × 0.10 × 0.10 mm

#### Data collection

Bruker SMART APEX CCD area-detector diffractometer
Absorption correction: multi-scan (**SADABS**; Bruker, 2003 ▶) *T*_min_ = 0.610, *T*_max_ = 0.862
12273 measured reflections
3105 independent reflections
2854 reflections with *I* > 2σ(*I*)
*R*_int_ = 0.024

#### Refinement

*R*[*F* ^2^ > 2σ(*F* ^2^)] = 0.020
*wR*(*F* ^2^) = 0.052
*S* = 1.07
3105 reflections
191 parameters
H-atom parameters constrained
Δρ_max_ = 1.36 e Å^−3^
Δρ_min_ = −0.48 e Å^−3^

### 

Data collection: *SMART* (Bruker, 2003 ▶); cell refinement: *SAINT* (Bruker, 2003 ▶); data reduction: *SAINT*; program(s) used to solve structure: *SHELXS97* (Sheldrick, 2008 ▶); program(s) used to refine structure: *SHELXL97* (Sheldrick, 2008 ▶); molecular graphics: *ORTEP-3* (Farrugia, 1997 ▶); software used to prepare material for publication: *SHELXTL* (Sheldrick, 2008 ▶).

## Supplementary Material

Crystal structure: contains datablocks I, global. DOI: 10.1107/S160053680904238X/is2471sup1.cif

Structure factors: contains datablocks I. DOI: 10.1107/S160053680904238X/is2471Isup2.hkl

Additional supplementary materials:  crystallographic information; 3D view; checkCIF report

## Figures and Tables

**Fig. 1.**
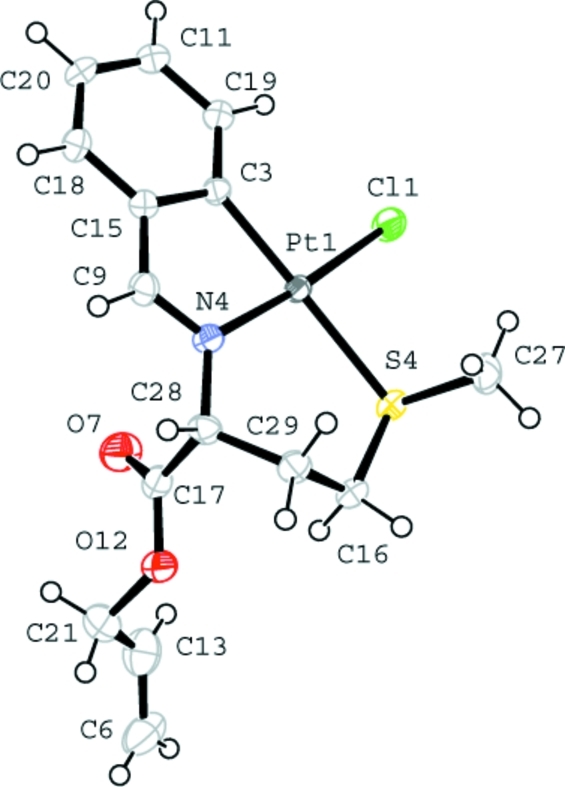
ORTEP drawing of (RC,SS)-1 with 50% probability displacement.

**Fig. 2.**
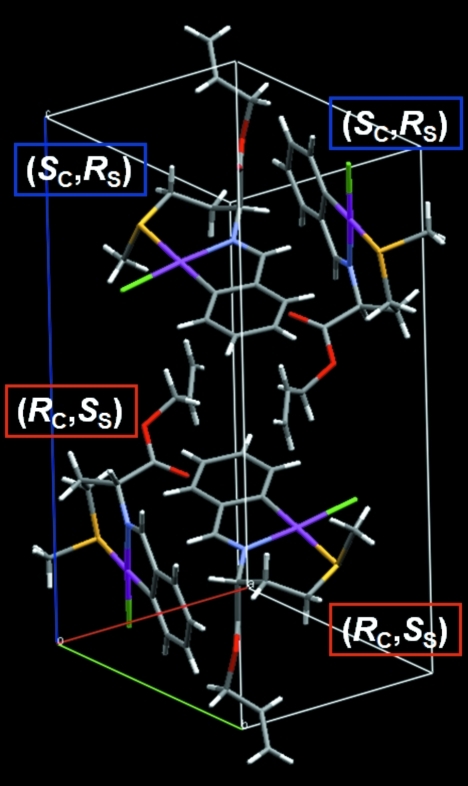
The packing structure of (1). Hydrogen atoms are omitted for clarity.

**Fig. 3.**
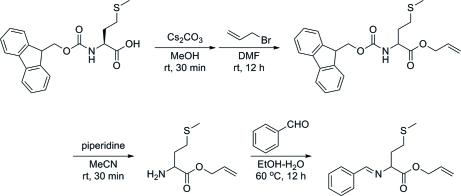
Synthetic scheme of the amino acid-derived ligand for 1.

## supplementary crystallographic information

### Comment

Research on cyclometallated complexes (Capapé *et al.*, 2005; Riera *et al.*, 2000) has been focused because of their fundamental applications as luminescent materials (Caubet *et al.*, 2003) and catalysts for a range of cross-coupling reactions. Amino acids, possessing natural chiral backbone and strong coordinating groups, are one of the potent candidates for designing stable cyclometallated complexes with highly controlled stereo-structure (Severin *et al.*, 1998). Here, we report the crystal structure of the platinum (II) complex (1), containing chiral methionine-derived *C*,*N*,*S*-terdentate ligand.

The crystal structure of title compound (1) is presented in Fig. 1. The Pt(II) atom is tetracoordinated in a square-planar environment by a Cl atom and C, N, S atoms from benzylidene methionine ester ligand. In the [Pt (C_15_H_18_NO_2_S)Cl] (1), S atoms showed opposite chiral configurations to the α-carbon of methionine for reducing steric repulsion between methyl and allyl ester groups (Fig. 2).

### Experimental

The methionine-derived ligand was synthesized from three step reactions, esterification, deprotection, condensation using Fmoc-*L*-methionine as a starting material (Fig. 3). A suspension of *cis*-[PtCl_2_(PhCN)_2_] and methionine-derived ligand was refluxed under nitrogen atmosphere for 3 h. The crude mixture was purified by SiO_2_ flash column chromatography. Single crystals for X-ray analyses were obtained from a CH_2_Cl_2_ solution.

### Refinement

H atoms were positioned geometrically and refined as riding atoms, with C_arene_—H and C_allyl_—H = 0.93 Å, C_methyl_—H = 0.96 Å, C_alkyl_—H = 0.97Å and C_α_—H = 0.98 Å, and with *U*_iso_(H) = 1.2*U*_eq_(C_arene_, C_alkyl_) and *U*_iso_(H) = 1.5*U*_eq_(C_methyl_). Electron density synthesis with coefficients *F_o_*—Fc: Highest peak 1.36 at 0.0428, 0.4390, 0.1909 (0.86 Å from Pt1)

### Crystal data

**Table:**

[Pt(C_15_H_18_NO_2_S)Cl]	*F*(000) = 968
*M**_r_* = 506.90	*D*_x_ = 2.098 Mg m^−^^3^
Monoclinic, *P*2_1_/*n*	Mo *K*α radiation, λ = 0.71073 Å
Hall symbol: -P 2yn	Cell parameters from 1024 reflections
*a* = 8.6000 (13) Å	θ = 2.8–26.0°
*b* = 9.5093 (14) Å	µ = 9.04 mm^−^^1^
*c* = 19.679 (3) Å	*T* = 180 K
β = 94.398 (2)°	Prism, orange
*V* = 1604.6 (4) Å^3^	0.50 × 0.10 × 0.10 mm
*Z* = 4	

### Data collection

**Table:**

Bruker SMART APEX CCD area-detector diffractometer	3105 independent reflections
Radiation source: fine-focus sealed tube	2854 reflections with *I* > 2σ(*I*)
graphite	*R*_int_ = 0.024
ω scans	θ_max_ = 26.0°, θ_min_ = 2.4°
Absorption correction: multi-scan (*SADABS*; Bruker, 2003)	*h* = −9→10
*T*_min_ = 0.610, *T*_max_ = 0.862	*k* = −11→11
12273 measured reflections	*l* = −24→24

### Refinement

**Table:**

Refinement on *F*^2^	Primary atom site location: structure-invariant direct methods
Least-squares matrix: full	Secondary atom site location: difference Fourier map
*R*[*F*^2^ > 2σ(*F*^2^)] = 0.020	Hydrogen site location: inferred from neighbouring sites
*wR*(*F*^2^) = 0.052	H-atom parameters constrained
*S* = 1.07	*w* = 1/[σ^2^(*F*_o_^2^) + (0.033*P*)^2^ + 0.6405*P*] where *P* = (*F*_o_^2^ + 2*F*_c_^2^)/3
3105 reflections	(Δ/σ)_max_ = 0.003
191 parameters	Δρ_max_ = 1.36 e Å^−^^3^
0 restraints	Δρ_min_ = −0.47 e Å^−^^3^

### Special details

**Table:**

Geometry. All e.s.d.'s (except the e.s.d. in the dihedral angle between two l.s. planes) are estimated using the full covariance matrix. The cell e.s.d.'s are taken into account individually in the estimation of e.s.d.'s in distances, angles and torsion angles; correlations between e.s.d.'s in cell parameters are only used when they are defined by crystal symmetry. An approximate (isotropic) treatment of cell e.s.d.'s is used for estimating e.s.d.'s involving l.s. planes.
Refinement. Refinement of *F*^2^ against ALL reflections. The weighted *R*-factor *wR* and goodness of fit *S* are based on *F*^2^, conventional *R*-factors *R* are based on *F*, with *F* set to zero for negative *F*^2^. The threshold expression of *F*^2^ > σ(*F*^2^) is used only for calculating *R*-factors(gt) *etc*. and is not relevant to the choice of reflections for refinement. *R*-factors based on *F*^2^ are statistically about twice as large as those based on *F*, and *R*- factors based on ALL data will be even larger.

### Fractional atomic coordinates and isotropic or equivalent isotropic displacement parameters (Å^2^)

**Table:**

	*x*	*y*	*z*	*U*_iso_*/*U*_eq_	
Pt1	0.456604 (14)	0.848674 (12)	0.309063 (6)	0.01942 (6)	
S4	0.63428 (10)	0.87893 (9)	0.22448 (5)	0.02394 (18)	
Cl1	0.60649 (11)	1.00955 (10)	0.37235 (5)	0.0338 (2)	
C3	0.3033 (4)	0.8222 (3)	0.37990 (18)	0.0224 (7)	
N4	0.3141 (3)	0.7078 (3)	0.26035 (14)	0.0222 (6)	
C6	0.1849 (5)	0.9473 (5)	−0.0659 (2)	0.0518 (12)	
H6A	0.1530	0.8793	−0.0980	0.062*	
H6B	0.2203	1.0341	−0.0801	0.062*	
O7	0.1383 (4)	0.8499 (3)	0.16257 (16)	0.0423 (7)	
C9	0.2011 (4)	0.6636 (3)	0.29340 (19)	0.0258 (8)	
H9	0.1318	0.5959	0.2752	0.031*	
C11	0.1695 (5)	0.8600 (4)	0.4821 (2)	0.0331 (9)	
H11	0.1639	0.9067	0.5234	0.040*	
O12	0.2371 (3)	0.7266 (3)	0.07819 (13)	0.0377 (6)	
C13	0.1798 (5)	0.9200 (5)	0.0011 (3)	0.0497 (11)	
H13	0.2122	0.9893	0.0324	0.060*	
C15	0.1855 (4)	0.7230 (3)	0.36017 (17)	0.0242 (7)	
C16	0.5768 (4)	0.7677 (4)	0.15222 (17)	0.0277 (7)	
H16A	0.5139	0.8229	0.1190	0.033*	
H16B	0.6699	0.7388	0.1311	0.033*	
C17	0.2223 (4)	0.7597 (4)	0.14303 (18)	0.0292 (8)	
C18	0.0640 (4)	0.6922 (4)	0.40057 (19)	0.0322 (8)	
H18	−0.0100	0.6246	0.3868	0.039*	
C19	0.2922 (4)	0.8882 (4)	0.44249 (18)	0.0274 (7)	
H19	0.3685	0.9524	0.4580	0.033*	
C20	0.0548 (4)	0.7633 (4)	0.46131 (18)	0.0366 (9)	
H20	−0.0277	0.7465	0.4881	0.044*	
C21	0.1259 (5)	0.7871 (5)	0.0261 (2)	0.0469 (11)	
H21A	0.1100	0.7217	−0.0116	0.056*	
H21B	0.0264	0.8009	0.0453	0.056*	
C27	0.8092 (4)	0.7906 (4)	0.2577 (2)	0.0355 (9)	
H27A	0.7881	0.6922	0.2627	0.053*	
H27B	0.8434	0.8298	0.3012	0.053*	
H27C	0.8892	0.8029	0.2268	0.053*	
C28	0.3198 (4)	0.6605 (3)	0.18980 (18)	0.0246 (8)	
H28	0.2675	0.5689	0.1867	0.030*	
C29	0.4852 (4)	0.6366 (3)	0.16894 (19)	0.0270 (8)	
H29A	0.5436	0.5870	0.2056	0.032*	
H29B	0.4793	0.5756	0.1293	0.032*	

### Atomic displacement parameters (Å^2^)

**Table:**

	*U*^11^	*U*^22^	*U*^33^	*U*^12^	*U*^13^	*U*^23^
Pt1	0.01859 (9)	0.02131 (9)	0.01877 (9)	−0.00125 (4)	0.00404 (6)	−0.00029 (4)
S4	0.0227 (4)	0.0254 (4)	0.0247 (5)	0.0004 (3)	0.0077 (3)	0.0000 (3)
Cl1	0.0320 (5)	0.0411 (5)	0.0291 (5)	−0.0148 (4)	0.0066 (4)	−0.0086 (4)
C3	0.0202 (18)	0.0253 (16)	0.0217 (18)	−0.0021 (13)	0.0012 (14)	0.0065 (13)
N4	0.0247 (15)	0.0216 (14)	0.0206 (14)	−0.0022 (11)	0.0042 (11)	−0.0008 (11)
C6	0.046 (3)	0.062 (3)	0.050 (3)	0.009 (2)	0.020 (2)	0.018 (2)
O7	0.0392 (17)	0.0494 (18)	0.0387 (17)	0.0171 (13)	0.0061 (14)	0.0016 (12)
C9	0.0243 (19)	0.0242 (18)	0.029 (2)	−0.0058 (13)	0.0012 (15)	−0.0004 (13)
C11	0.030 (2)	0.047 (2)	0.022 (2)	−0.0010 (16)	0.0050 (16)	−0.0026 (15)
O12	0.0289 (14)	0.0599 (18)	0.0244 (13)	0.0047 (13)	0.0030 (11)	0.0035 (12)
C13	0.036 (2)	0.052 (3)	0.062 (3)	0.007 (2)	0.005 (2)	0.001 (2)
C15	0.0224 (17)	0.0290 (18)	0.0211 (17)	−0.0015 (13)	0.0013 (13)	0.0021 (13)
C16	0.0244 (18)	0.036 (2)	0.0236 (17)	0.0017 (15)	0.0079 (14)	−0.0030 (15)
C17	0.0224 (18)	0.037 (2)	0.0287 (19)	−0.0002 (15)	0.0044 (14)	0.0039 (16)
C18	0.024 (2)	0.042 (2)	0.030 (2)	−0.0109 (16)	0.0030 (15)	0.0034 (17)
C19	0.0253 (19)	0.0330 (18)	0.0240 (18)	−0.0018 (15)	0.0021 (14)	−0.0021 (15)
C20	0.028 (2)	0.059 (3)	0.0234 (19)	−0.0062 (18)	0.0097 (15)	0.0058 (17)
C21	0.036 (2)	0.071 (3)	0.033 (2)	0.000 (2)	−0.0041 (18)	0.009 (2)
C27	0.0222 (19)	0.043 (2)	0.041 (2)	0.0063 (16)	0.0015 (16)	−0.0047 (18)
C28	0.029 (2)	0.0233 (18)	0.0219 (18)	−0.0019 (13)	0.0018 (15)	−0.0044 (12)
C29	0.027 (2)	0.0287 (18)	0.0255 (19)	0.0064 (14)	0.0037 (15)	−0.0075 (14)

### Geometric parameters (Å, °)

**Table:**

Pt1—C3	2.006 (4)	C13—C21	1.446 (7)
Pt1—N4	2.009 (3)	C13—H13	0.9300
Pt1—Cl1	2.3037 (9)	C15—C18	1.392 (5)
Pt1—S4	2.3618 (9)	C16—C29	1.524 (5)
S4—C27	1.801 (4)	C16—H16A	0.9700
S4—C16	1.810 (3)	C16—H16B	0.9700
C3—C19	1.392 (5)	C17—C28	1.524 (5)
C3—C15	1.417 (5)	C18—C20	1.381 (5)
N4—C9	1.281 (4)	C18—H18	0.9300
N4—C28	1.464 (4)	C19—H19	0.9300
C6—C13	1.348 (6)	C20—H20	0.9300
C6—H6A	0.9300	C21—H21A	0.9700
C6—H6B	0.9300	C21—H21B	0.9700
O7—C17	1.204 (4)	C27—H27A	0.9600
C9—C15	1.446 (5)	C27—H27B	0.9600
C9—H9	0.9300	C27—H27C	0.9600
C11—C19	1.386 (5)	C28—C29	1.528 (5)
C11—C20	1.387 (5)	C28—H28	0.9800
C11—H11	0.9300	C29—H29A	0.9700
O12—C17	1.330 (4)	C29—H29B	0.9700
O12—C21	1.464 (5)		
			
C3—Pt1—N4	80.72 (13)	O7—C17—O12	125.4 (3)
C3—Pt1—Cl1	94.46 (10)	O7—C17—C28	124.4 (3)
N4—Pt1—Cl1	175.18 (8)	O12—C17—C28	110.1 (3)
C3—Pt1—S4	179.20 (10)	C20—C18—C15	119.1 (3)
N4—Pt1—S4	98.56 (8)	C20—C18—H18	120.4
Cl1—Pt1—S4	86.26 (3)	C15—C18—H18	120.4
C27—S4—C16	100.53 (18)	C11—C19—C3	121.2 (3)
C27—S4—Pt1	104.77 (14)	C11—C19—H19	119.4
C16—S4—Pt1	109.27 (12)	C3—C19—H19	119.4
C19—C3—C15	116.5 (3)	C18—C20—C11	119.6 (3)
C19—C3—Pt1	130.7 (3)	C18—C20—H20	120.2
C15—C3—Pt1	112.8 (2)	C11—C20—H20	120.2
C9—N4—C28	117.6 (3)	C13—C21—O12	111.9 (4)
C9—N4—Pt1	115.8 (2)	C13—C21—H21A	109.2
C28—N4—Pt1	126.4 (2)	O12—C21—H21A	109.2
C13—C6—H6A	120.0	C13—C21—H21B	109.2
C13—C6—H6B	120.0	O12—C21—H21B	109.2
H6A—C6—H6B	120.0	H21A—C21—H21B	107.9
N4—C9—C15	117.4 (3)	S4—C27—H27A	109.5
N4—C9—H9	121.3	S4—C27—H27B	109.5
C15—C9—H9	121.3	H27A—C27—H27B	109.5
C19—C11—C20	121.1 (4)	S4—C27—H27C	109.5
C19—C11—H11	119.4	H27A—C27—H27C	109.5
C20—C11—H11	119.4	H27B—C27—H27C	109.5
C17—O12—C21	118.2 (3)	N4—C28—C17	109.0 (3)
C6—C13—C21	122.5 (5)	N4—C28—C29	113.6 (3)
C6—C13—H13	118.8	C17—C28—C29	114.2 (3)
C21—C13—H13	118.8	N4—C28—H28	106.5
C18—C15—C3	122.4 (3)	C17—C28—H28	106.5
C18—C15—C9	124.2 (3)	C29—C28—H28	106.5
C3—C15—C9	113.3 (3)	C16—C29—C28	116.4 (3)
C29—C16—S4	115.0 (2)	C16—C29—H29A	108.2
C29—C16—H16A	108.5	C28—C29—H29A	108.2
S4—C16—H16A	108.5	C16—C29—H29B	108.2
C29—C16—H16B	108.5	C28—C29—H29B	108.2
S4—C16—H16B	108.5	H29A—C29—H29B	107.4
H16A—C16—H16B	107.5		
			
N4—Pt1—S4—C27	−107.41 (16)	C21—O12—C17—C28	165.9 (3)
Cl1—Pt1—S4—C27	72.70 (14)	C3—C15—C18—C20	1.8 (6)
Cl1—Pt1—S4—C16	179.70 (13)	C9—C15—C18—C20	−175.2 (3)
N4—Pt1—C3—C19	−176.3 (4)	C20—C11—C19—C3	1.1 (6)
Cl1—Pt1—C3—C19	3.6 (3)	C15—C3—C19—C11	−1.6 (5)
N4—Pt1—C3—C15	1.1 (2)	Pt1—C3—C19—C11	175.8 (3)
Cl1—Pt1—C3—C15	−179.0 (2)	C15—C18—C20—C11	−2.4 (6)
C3—Pt1—N4—C9	−1.8 (3)	C19—C11—C20—C18	1.0 (6)
S4—Pt1—N4—C9	178.5 (2)	C6—C13—C21—O12	128.9 (4)
C3—Pt1—N4—C28	172.9 (3)	C17—O12—C21—C13	90.1 (5)
S4—Pt1—N4—C28	−6.8 (3)	C9—N4—C28—C17	86.9 (4)
C28—N4—C9—C15	−173.0 (3)	Pt1—N4—C28—C17	−87.7 (3)
Pt1—N4—C9—C15	2.2 (4)	C9—N4—C28—C29	−144.4 (3)
C19—C3—C15—C18	0.1 (5)	Pt1—N4—C28—C29	40.9 (4)
Pt1—C3—C15—C18	−177.7 (3)	O7—C17—C28—N4	−9.1 (5)
C19—C3—C15—C9	177.5 (3)	O12—C17—C28—N4	174.3 (3)
Pt1—C3—C15—C9	−0.3 (4)	O7—C17—C28—C29	−137.4 (4)
N4—C9—C15—C18	176.1 (3)	O12—C17—C28—C29	46.0 (4)
N4—C9—C15—C3	−1.2 (4)	S4—C16—C29—C28	69.7 (4)
C27—S4—C16—C29	83.2 (3)	N4—C28—C29—C16	−78.1 (4)
Pt1—S4—C16—C29	−26.7 (3)	C17—C28—C29—C16	47.8 (4)
C21—O12—C17—O7	−10.6 (6)		
